# Precocious obesity predisposes the development of more severe cisplatin-induced acute kidney injury in young adult mice

**DOI:** 10.1371/journal.pone.0174721

**Published:** 2017-03-30

**Authors:** Rosemara S. Ribeiro, Clevia S. Passos, Antônio S. Novaes, Edgar Maquigussa, Maria A. Glória, Iria Visoná, Olinda Ykuta, Lila M. Oyama, Mirian A. Boim

**Affiliations:** 1 Renal Division, Department of Medicine–Federal University of São Paulo, São Paulo, Brazil; 2 Pathology Department–Federal University of São Paulo, São Paulo, Brazil; 3 Nutrition Physiology–Department of Physiology—Federal University of São Paulo, São Paulo, Brazil; National Institutes of Health, UNITED STATES

## Abstract

Obesity and its consequences can damage the kidney over time. However, less is known about the impact of developing overweight/obesity during childhood on the kidney in adulthood and the renal impact of a superimposed acute kidney injury (AKI). This study evaluated the effect of obesity induced by a high-fat diet initiated soon after weaning on the adult life of mice and their response to superimposed nephrotoxic effects of cisplatin. C57BL/6 post-weaning mice (3 weeks old) were divided into a control group (CT, n = 12) and a high-fat diet group (HF, n = 12). After 9 weeks, animals were further divided into the following groups: CT, CT treated with a single dose of cisplatin (CTCis, 20 mg/kg, i.p.), HF and HF treated with cisplatin (HFCis). The HF group exhibited higher body weight gain compatible with a moderate obesity. Obese mice presented increased visceral adiposity, hyperkalemia, sodium retention, glomerular hyperfiltration and proteinuria, without any significant changes in blood pressure and glycemia. AKI induced by cisplatin was exacerbated in obese animals with a 92% reduction in the GFR versus a 31% decrease in the CTCis group; this sharp decline resulted in severely elevated serum creatinine and urea levels. Acute tubular necrosis induced by cisplatin was worsened in obese mice. The HFCis group exhibited robust systemic and intrarenal inflammation that was significantly higher than that in the CTCis group; the HFCis group also showed a higher degree of renal oxidative stress. In conclusion, the moderate degree of obesity induced shortly after weaning resulted in mild early renal alterations, however, obese young animals were prone to develop a much more severe AKI induced by cisplatin.

## Introduction

The rates of adult and childhood obesity are increasing worldwide, with 41 million children affected by overweight or obesity [1, 2]. Obesity predisposes individuals to insulin resistance, type 2 diabetes, dyslipidemia, hypertension, inflammation, oxidative stress, liver and renal diseases during childhood or adulthood [3].

Obesity is a risk factor for the development and progression of kidney disease in both adults and children [4, 5]. Despite the increased glomerular filtration rate (GFR) caused by obesity, the mesangial expansion, basement membrane thickening, podocyte stress and inflammation can lead to glomerulosclerosis and tubulointerstitial injury over time [6, 7]. Abnormal albumin excretion and hyperfiltration have been observed in obese children and adolescents even in the absence of diabetes [8]. Thus, obesity constitutes a risk factor for the development of chronic kidney disease in adult individuals [9, 10]; however, less is known on the impact of the onset of obesity early during childhood on the renal function in those respective adults.

Renal hemodynamic alterations, inflammation and oxidative stress are often observed in obese individuals, and although these manifestations may be aggravated by many associated factors such as hypertension and diabetes, the impact of acute kidney injury superimposed to obese individuals is unknown.

Acute kidney injury (AKI) is a serious pathological condition triggered by many factors including nephrotoxic drugs. Cisplatin is one of the most effective and widely used chemotherapeutic agent for the treatment of a variety of solid tumors; however, its major side effect is nephrotoxicity [11, 12]. Cisplatin-induced AKI involves proximal tubular injury with tubular necrosis, oxidative stress, inflammation, and vascular injury [13]. There is also concomitant activation of multiple proinflammatory cytokines and infiltration of inflammatory cells into the kidney [14].

Obesity is associated with more severe AKI in hospitalized and critically ill patients, and this association is dependent on the illness severity and obesity-associated comorbidities [15, 16]. However, the impact of the AKI superimposed to moderately obese healthy individual is unknown.

The AKI model induced by cisplatin was chosen in this study because there is a recognizable relationship between obesity and cancer [17, 18] and because after a single dose of cisplatin, approximately one-third of the patients develop nephrotoxicity [19]. The objectives of this study were to evaluate the consequences of obesity induced shortly after weaning on adult systemic parameters and renal function in mice and to determine whether this model of obesity predisposes mice to a more aggressive nephrotoxic response induced by cisplatin.

## Material and methods

This study was approved by the Ethics Committee for use of animals at the Federal University of São Paulo (process N° 7235300915). Young male (3 weeks old; post-weaning) wild type C57BL/6 mice were purchased from the Center for the Development of Experimental Animal Models at the Federal University of São Paulo. The animals were maintained under a 12-h light/12-h dark cycle under constant room temperature (22±1°C) with free access to food and tap water. On the first day after weaning, animals were randomly divided into two experimental groups: the control group, which was fed a standard mouse diet (CT, n = 12) and the second group that was fed a high-fat diet (HF, n = 12). The standard diet (Nuvilab, Parana, Brazil) contained 22 g % of protein, 56 g % of carbohydrate, 4 g % of fat and 3.80 Kcal/g. The high-fat diet (Research Diets, D12451, New Jersey, USA), contained 24 g of protein (20 kcal%), 41 g of carbohydrate (35 kcal%), 24 g of fat (45 kcal%) and 4.73 Kcal/g. After 9 weeks under their respective nutritional regimen, mice were fasted during 12 hr and then subjected to tail vein blood collection for glycemia determination (Accu-Chek Performa Nano, Roche Diagnostics, Indianapolis, USA). Systolic blood pressure was estimated by pletismography (PowerLab, ADInstruments, Bella Vista, Australia). Food intake was estimated by measuring the food consumed per group of 6 mice/cage over a 24-hr period. Then, animals were further subdivided as follows: control diet animals receiving vehicle (CT, n = 6); control diet animals treated with cisplatin (CTCis, n = 6); HF diet animals receiving vehicle (HF, n = 6) and HF diet animals treated with cisplatin (HFCis), n = 6). Either cisplatin (20 mg/kg, Sigma-Aldrich, Missouri, USA) or vehicle (saline solution) were administered by a single intraperitoneal injection. At 24 hr after cisplatin or vehicle administration, the animals were transferred to metabolic cages with free access to tap water to allow for 24-hr urine collection and the determination of individual water intake. After the metabolic cage study, the animals were returned to collective cages for an additional 48 hr. At 72 hr after cisplatin administration, the animals were anesthetized with ketamine (70 mg/kg) and xylazine (5 mg/kg), and blood samples were collected via cardiac puncture. Then, the animals were euthanized by cervical dislocation. Visceral fat (mesenteric, epididymis and retroperitoneal) were removed and weighed on an analytical balance (AX 200, Shimadzu, Tokyo, Japan) and the adiposity index was calculated by considering the total mass of the visceral fat corrected by body mass. Both kidneys were removed, and the right kidney was immediately frozen in liquid nitrogen and stored at −80°C for future use. The left kidney was immediately fixed in 10% buffered formalin for histological analysis.

### Renal function parameters

Serum and urinary creatinine and urea concentrations as well as urinary protein content were determined by using commercial kits (Labtest, Minas Gerais, Brazil) according to the manufacturer instructions. Serum and urinary sodium and potassium levels were determined using a flame photometer (B462, Micronal, São Paulo, Brazil).

### Gene expression of cytokines and oxidative stress markers

Quantification of the mRNA levels of inflammatory and oxidative stress markers was performed by using real-time RT-PCR. Total RNA was purified from the renal tissue using the phenol and guanidine isothiocyanate-cesium chloride method (TRIzol, Life Technologies, Carlsbad, CA, USA), and total amount was quantified in a spectrophotometer (NanoVue, GE Healthcare Life Science, UK). cDNA was synthetized from 2 μg of total RNA using a mixture containing 0.5 mg/ml oligo (dT), 10 mM dithiothreitol (DTT), 0.5 mM deoxynucleoside triphosphates (Amersham Pharmacia Biotech, Uppsala, Sweden), and 200 U of reverse transcriptase enzyme (SuperScript RT, ThermoScientific, USA) in the presence of 1 U DNase (Promega, Madison, USA) to eliminate genomic DNA contamination. The mRNA expression levels were estimated using quantitative real-time PCR (QuantStudio Flex 7 Real-Time PCR System, Applied Biosystems, Carlsbad, CA, USA). Real-time RT-PCR was performed using the SYBR® system (Applied Biosystems, Carlsbad, CA, USA). Primers for amplification were designed based on GenBank sequences (Missouri, USA) for tumor necrosis factor alpha (TNF-α), interleukin 6 (IL-6), superoxide dismutase 1 (SOD1), NADPH oxidase activating enzyme (gp91phox) and β-actin and were synthesized by Sigma-Aldrich. The following forward and reverse primers, respectively, were used: TNF-α (CTATGTCTCAGCCTCTTCTC and CATTTGGGAACTTCTCATCC); IL-6 (AAGAAATGATGGATGCTACC and GAGTTTCTGTATCTCTCTGAAG); SOD-1 (CACTCTAAGAAACATGGTGG and GATCACACGATCTTCAATGG); gp91phox (CCTCTATGCCAACACAGTGC and ACATCTGCTGGAAGGTGGAC); β-actin (CCTCTATGCCAACACAGTGC and ACATCTGCTGGAAGGTGGAC). The comparative CT method (ΔΔCT) was employed to quantify gene expression, and the relative mRNA quantification was calculated as 2^ΔΔCT^. The mRNA expression levels were normalized to β-actin expression, which was used as an endogenous control.

### Serum and renal cytokines quantification

An immunoassay panel (Milliplex Mouse cytokine/chemokine, EMD Millipore, Massachusetts, USA) for IL-6, IL-10 and TNF-α was used to analyze the inflammatory profile in serum and renal tissue. Kidney proteins were extracted using RIPA lysis buffer, and the protein was quantified according to manufacturer instructions. The analysis was performed by a multiplex detection system (Luminex 100/200, EMD Millipore, Massachusetts, USA). The results were expressed as pg/mL for serum and pg/mg protein for renal tissue.

### Lipid peroxidation in kidney

The oxidative degradation of lipids in cell membranes resulting in cell damage was estimated by quantifying malondialdehyde. Approximately 500 μL of protein extract from the kidney was added to 500 μL of a working solution comprising 9 g trichloroacetic acid, 0.225 g thiobarbituric acid, 1.74 μL of hydrochloric acid and 60 mL distilled water. The mixture was heated in a 100°C water bath for 30 minutes followed by incubation on ice for 20 minutes, after which the contents were centrifuged at 9.500 g for 10 minutes. The standard curve was constructed using malondialdehyde at concentrations of 0.625, 1.25, 2.5, 5 and 10 μmol and measured on a spectrophotometer (EON, BioTek, Vermont, USA) at 532 nm to determine the concentration of malondialdehyde in the samples. The results were expressed as μmol/mg protein.

### Protein expression

Relative quantification of the renal proteins nephrin, podocin, glutathione peroxidase and tubulin (used as an endogenous control) was performed by using the western blot technique. Renal tissue was homogenized in RIPA lysis buffer (pH 8) containing 50 mM TRIS (Sigma-Aldrich, Missouri, USA), 150 mM sodium chloride (Labsynth, São Paulo, Brazil), 1.0% NP-40 (Bio-Rad Laboratories, Hercules, CA, USA), 0.5% sodium deoxycholate (Sigma-Aldrich, Missouri, USA), and 0.1% sodium dodecyl sulfate (Sigma-Aldrich, Missouri, USA) containing a protease inhibitor cocktail (Sigma-Aldrich, Missouri, USA). Total protein was quantified by the Lowry method using a commercial kit (Protein assay reagent, Bio-Rad Laboratories, Hercules, USA) according to manufacturer instructions. Protein samples (50 μg) were separated according to size in a 12% gel via SDS-PAGE and electroblotted onto nitrocellulose membranes (GE Healthcare Life Sciences, UK) that were subsequently blocked for 1 hr at room temperature with 3% bovine serum albumin (Sigma-Aldrich, Missouri, USA) in Tris-buffered saline containing 0.05% Tween 20 (TBST, Bio-Rad Laboratories, Hercules, USA). The membrane blots were probed with the following primary antibodies overnight at 4°C in TBST buffer containing 3% bovine serum albumin: anti-nephrin antibody (Abcam, Cambridge, UK), 1:200 dilution; anti-podocin antibody (Santa Cruz Biotechnology, Dallas, USA), 1:500 dilution, anti-glutathione peroxidase (Abcam, Cambridge, UK), 1:1000 dilution and anti-acetylated tubulin antibody (Sigma-Aldrich, Missouri, USA), 1:30.000 dilution. Membranes were washed three times with TBST buffer and incubated with either an anti-rabbit (GE Healthcare Life Sciences, UK) or anti-mouse (Sigma-Aldrich, Missouri, USA) horseradish peroxidase-conjugated secondary antibody (1:30.000 dilution in TBST containing 3% bovine serum albumin) for 1 hr at room temperature. The protein bands were visualized by chemiluminescence (Alliance, UVITEC, Cambridge, UK) using a chemiluminescent reagent (Immobilon Western HRP substrate, EMD Millipore, Massachusetts, USA). The observed bands were quantified using UVIBAND image qualification software (UVITEC, Cambridge, UK) and expressed as a percentage of the tubulin expression, which was used as an endogenous control.

### Morphometric analysis of the glomeruli

PAS-stained renal cortex sections were used to estimate the glomerular area. Glomerular images were obtained using a light microscope (DM1000, Leica, Wetzlar, Germany), and only glomeruli containing a visible vascular or urinary pole were considered for the area measurements. Areas of Bowman’s capsule and the glomerular tuft were determined by using a computer-assisted image analyzer (AxioVision 4.6 Carl Zeiss Microscopy, Goettingen, Alemanha). Twenty glomeruli per slide were analyzed.

### Histological analyses

Formalin-fixed left kidneys were dehydrated, embedded in paraffin blocks, cut into 4-μm thick sections and stained with Periodic Acid Schiff (PAS).

### Apoptosis detection

Apoptosis was determined by TUNEL stain (ApopTag Peroxidase Kit, EMD Millipore, Massachusetts, USA), according to the manufacturer's instructions.

### Statistical analysis

The results are expressed as the mean±standard error. The data were analyzed by using SigmaStat software version 3.5. Student’s t-test was used to compare the results between the CT and HF groups. Two-way analysis of variance (ANOVA) followed by Tukey’s post hoc test was used to compare the effects of cisplatin in the CT and HF groups. Statistical significance was defined as p<0.05.

## Results

### Post-weaning high-fat diet induced moderate degree of obesity

**Fig 1:**
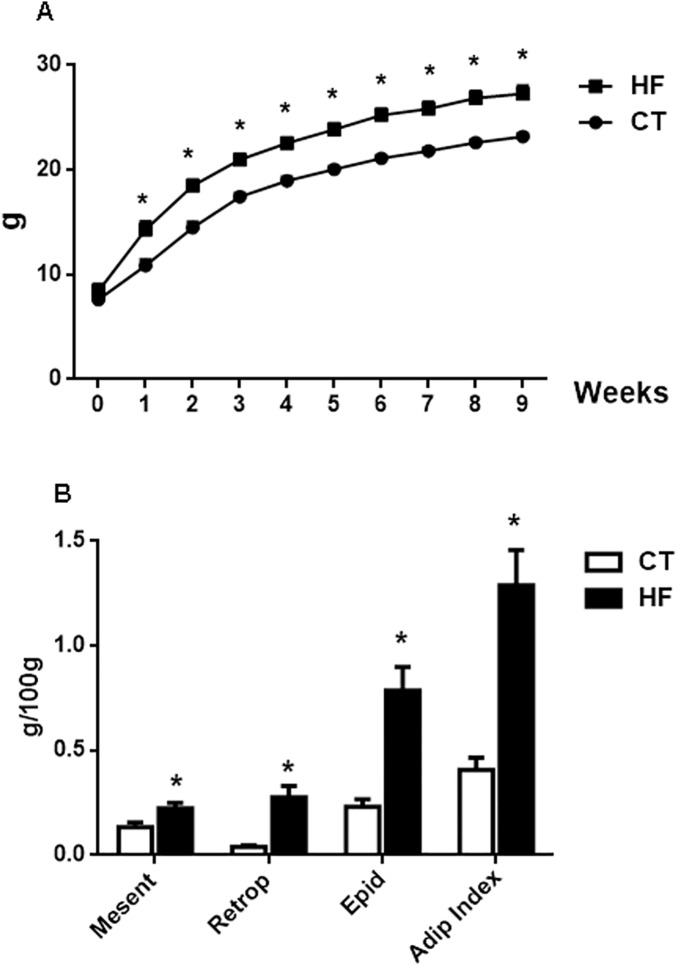
A: Body mass evolution in the control (CT) group fed standard chow and mice fed a high-fat (HF) diet (n = 12/group); B: Content of visceral, mesenteric, retroperitoneal and epididymal fat as well as the adiposity index; the values for visceral fat accumulation and adiposity index were determined in the CT and HF groups receiving vehicle (n = 6 per group); *p<0.05 vs CT.

Animals fed a HF diet exhibited a higher body mass gain from the first week after initiating HF diet consumption compared with CT animals (Fig 1A), and the body mass in the HF animals remained higher than that in the CT group throughout the 9-week period. The higher body mass gain in HF mice was characterized by an increase in the visceral fat content including mesenteric, retroperitoneal and epididymal fat, as well as an increased adiposity index (Fig 1B).

Table 1 shows the physiological parameters of the mice after 9 weeks of nutritional support. Food consumption by the HF animals was lower than that in animals fed with standard diet; however, the HF animals ingested more calories than the CT group, resulting in a body mass gain 25% higher than of animals in the CT group (p<0.05). The systolic blood pressure and fasting glucose were similar between both groups. Water intake by HF mice was reduced compared to CT animals; however, the urinary volume was unchanged.

**Table 1:** Physiological parameters.

Parameter	CT	HF
	(n = 12)	(n = 12)
Food Intake (g/day)	3.35±0.01	2.64±0.02*
Calorie intake (kcal/day)	11.83±0.01	12.58±0.09*
Body mass Gain (g)	12,70±0,55	16,08±1,20*
Systolic Blood Pressure (mmHg)	112±6.69	112±4.72
Fasting Serum glucose (mg/dL)	103±4.42	112±6.18
Water Intake (mL/day)	5.81±0.02	4.18±0.02*
Urinary Volume (mL/day)	1.56±0.25	2.03±0.48

Physiological parameters measured 9 weeks after nutrition support. Food intake per each animal was estimated by measuring the food consumption during 24 hr by a group of 6 animals. Serum glucose was measured after 12 hr fasting. Water intake and urinary volume were obtained in individual metabolic cage. p<0.05: *vs CT.

### Cisplatin induced additional visceral fat accumulation in obese mice

**Fig 2:**
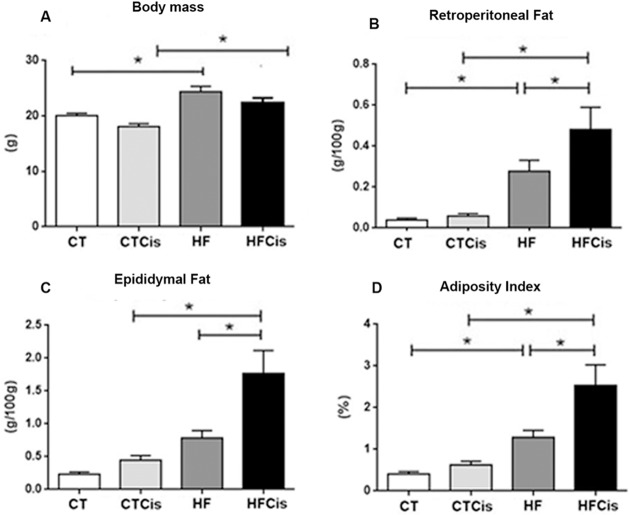
Body mass, visceral fat weight and adiposity index obtained 3 days after cisplatin or saline administration. The data are expressed as the mean ± SEM, n = 6 per group. *p<0.05.

There was a decrease in body mass 3 days after cisplatin administration in both groups, but, surprisingly, despite the reduction in the body mass, the HFCis group exhibited a significant increase in the visceral fat weight (retroperitoneal and epididymal) and in the adiposity index compared to CTCis animals (Fig 2).

### Renal morphological changes induced by obesity and cisplatin

**Fig 3:**
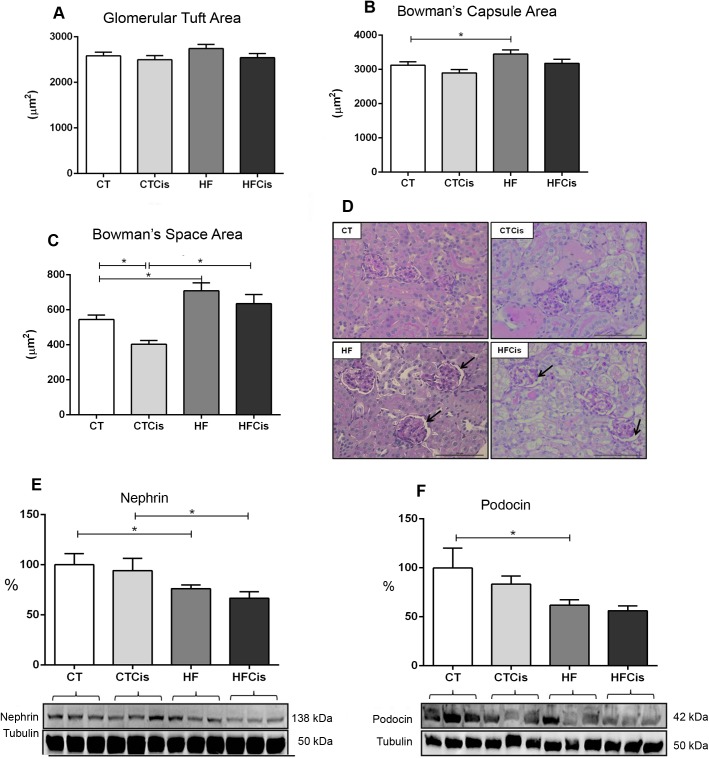
Morphometric analysis of the glomeruli. A: glomerular tuft area; B: Bowman’s capsule area; C: Bowman’s space area; D: representative PAS-stained cortex showing details of the Bowman’s space expansion in the HF and HFCis groups (arrows), 40x magnification, bar = 100 μm; E and F: podocin and nephrin protein expression levels with the respective representative western blotting membranes (n = 6 form each group). The data of morphometric analysis are expressed as the mean ± SEM, n = 4 per group, *p<0.05.

Kidneys from obese animals presented a well-preserved parenchyma; however, the glomeruli often presented an increased Bowman’s capsule area (Fig 3B), an expanded Bowman’s space (Fig 3C) with no significant alterations in the glomerular area (Fig 3A). Representative histological slide with expanded Bowman’s space is shown in Fig 3D. Obese mice presented significant reduction in the nephrin and podocin protein expressions which was additionally reduced by cisplatin. In contrast, cisplatin did not significantly changed both nephrin and podocin expressions in non-obese mice (Fig 3E and 3F).

### Effects of cisplatin were more severe in obese mice

Animals receiving cisplatin ingested less food compared to non-treated animals; however, the reduction in food consumption by obese mice was more severe, which indicated a debilitating condition in these animals. There was a decrease in water intake by animals treated with cisplatin in both the CT and HF groups; nevertheless, the urinary volume was not significantly changed by cisplatin (Table 2).

**Table 2:** Physiological parameters after cisplatin administration.

Parameter	CT	CTCis	HF	HFCis
	(n = 6)	(n = 6)	(n = 6)	(n = 6)
Water Intake (mL/day)	5.81±0.01	2.17±0.65*	4.18±0.02*	2.00±0.52^&^
Urinary Volume (mL/day)	1.56±0.25	1.44±0.19	2.03±0.48	1.28±0.10
Food Intake (g/day)	3.35±0.01	1.93±0.01*	2.64±0.01*	0.13±0.01^#,&^

Parameters were obtained in metabolic cage 24 hr after cisplatin or saline administration. p<0.05: * vs CT; ^#^ vs CTCis; ^&^ vs HF

**Fig 4:**
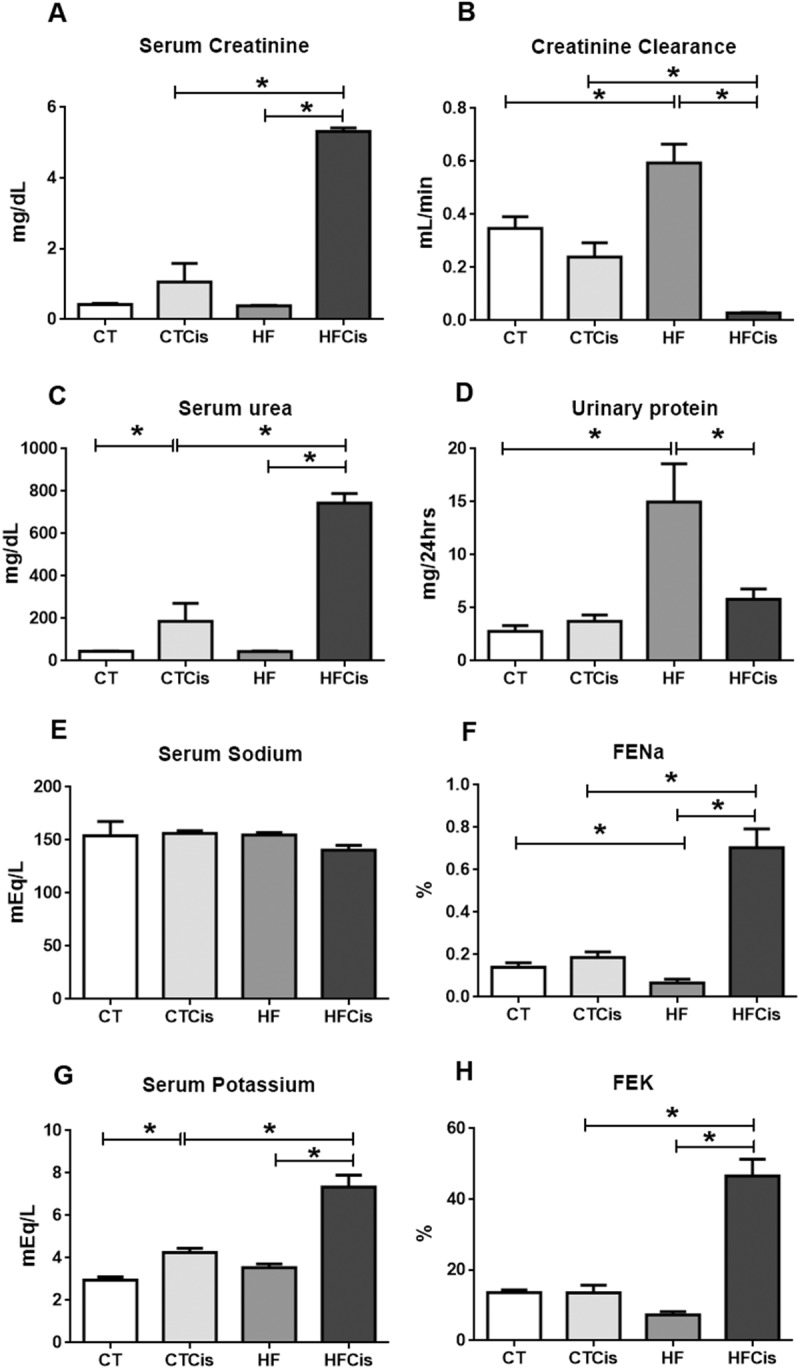
Renal function parameters. Serum levels of creatinine (A) and urea (C); B: creatinine clearance; D: urinary excretion of protein; E: serum sodium; F: fractional excretion of sodium; G: serum potassium; H: fractional excretion of potassium. The CT and HF groups received cisplatin vehicle or saline. The data are expressed as the mean ± SEM, n = 4–6 per group, *p<0.05.

As expected, cisplatin caused acute kidney injury characterized by increased serum levels of creatinine and mainly urea; however, the accumulation of serum creatinine and urea was exacerbated in obese animals, which had an approximately 10-fold increase in both parameters (Fig 4A and 4C). This result was the consequence of an impressive decline (91%) in the creatinine clearance in the HFCis group (Fig 4B). Obese animals presented significant proteinuria and, despite the rapid decline in creatinine clearance, the HFCis group exhibited decreased urinary protein excretion compared with the HF group (Fig 4D). Serum sodium remained unchanged in all groups, however, the fractional excretion of sodium (FENa) was reduced in obese mice which was significantly increased by cisplatin (Fig 4F). FEK was higher in the HFCis animals compared to the CTCis animals. In spite of the significant K^+^ loss, serum K^+^ was critically elevated in the HFCis group (Fig 4G–4H).

**Fig 5:**
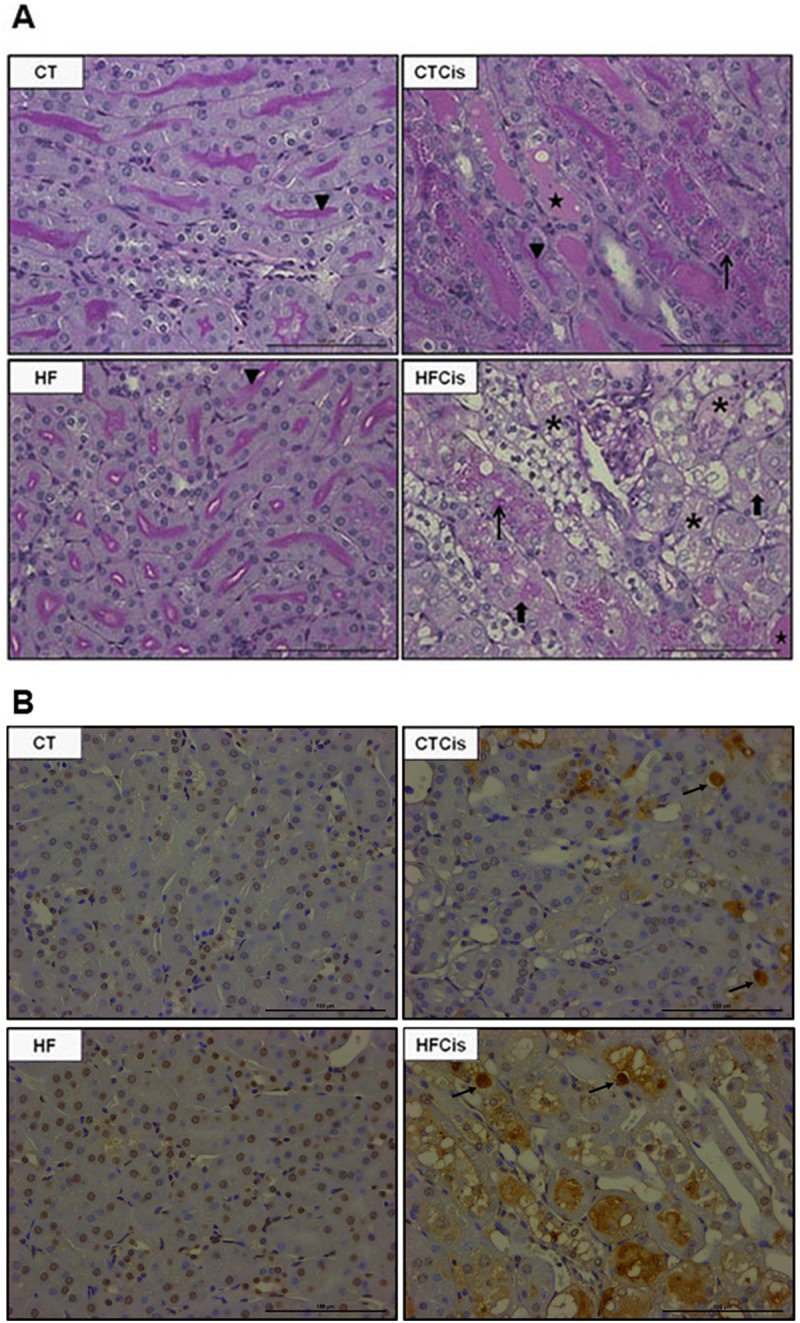
Tubular cell death analysis. A: Representative images of PAS-stained kidney sections. The CT and HF slides show the integrity of the renal parenchyma with a preserved border brush (triangle). The CTCis and HFCis animals exhibited degeneration and areas and necrotic cells characterized by hyaline casts (star), hyaline drops (thin arrow), brush border loss (large arrow) and disorganization of the tubular epithelium (asterisk). B: Representative images of TUNEL labelling characterizing apoptosis. 40x magnification and bars = 100 μm.

The renal parenchyma was preserved in vehicle-treated CT and HF animals (Fig 5A). In contrast, kidneys from the cisplatin-treated animals presented focal degenerative areas with necrotic tubules. The presence of hyaline casts, hyaline drops, brush border loss and tubular epithelium cell death was observed in both groups (Fig 5A), but the damage caused by cisplatin was more pronounced in obese animals, as indicated by the extensive tubular necrosis and areas with tissue disorganization. TUNEL technique estimating apoptosis is shown in Fig 5B. Apoptosis was detected in CT and HF animals treated with cisplatin (showed by arrows), however, the labelling was also detected in great extensions of necrotic areas, mainly in obese animals receiving cisplatin, hindering the visualization and quantification of apoptotic cells.

### Obesity was characterized by mild renal oxidative stress and systemic inflammation, which were potentiated by cisplatin

**Fig 6:**
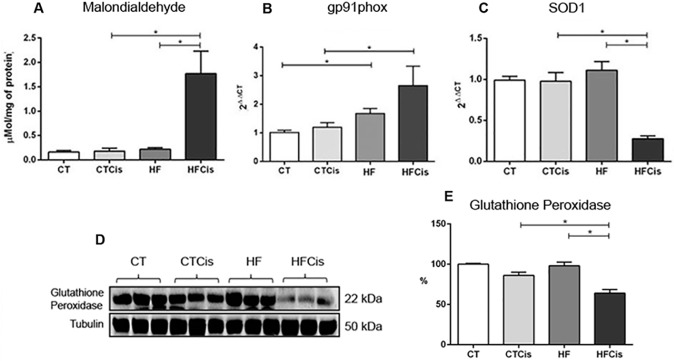
Renal expression of oxidative stress markers. A: Malondialdehyde, a marker of lipid peroxidation, was quantified by ELISA; B and C: the gene expression levels of the NADPH system enzyme gp91phox and the antioxidant enzyme SOD1 were determined by real-time RT-PCR. The mRNA levels were normalized to β-actin expression and quantified using the 2^ΔΔCT^ method. D-E: Representative western blot and densitometry analysis of glutathione peroxidase expression. The data are expressed as the mean ± SEM, n = 3–6 per group. *p<0.05.

Kidneys from obese animals presented a mild degree of oxidative stress as evidenced by an increase in the mRNA expression levels of gp91phox, a pro-oxidant enzyme, with no changes in the intrarenal levels of malondialdehyde (lipid peroxidation), of SOD1 mRNA expression levels or in the glutathione peroxidase protein levels (Fig 6A–6E). Surprisingly, none of the oxidative stress markers evaluated in the preset study was modified by cisplatin in CT group, however, all of them were significantly changed in obese animals receiving cisplatin.

**Fig 7:**
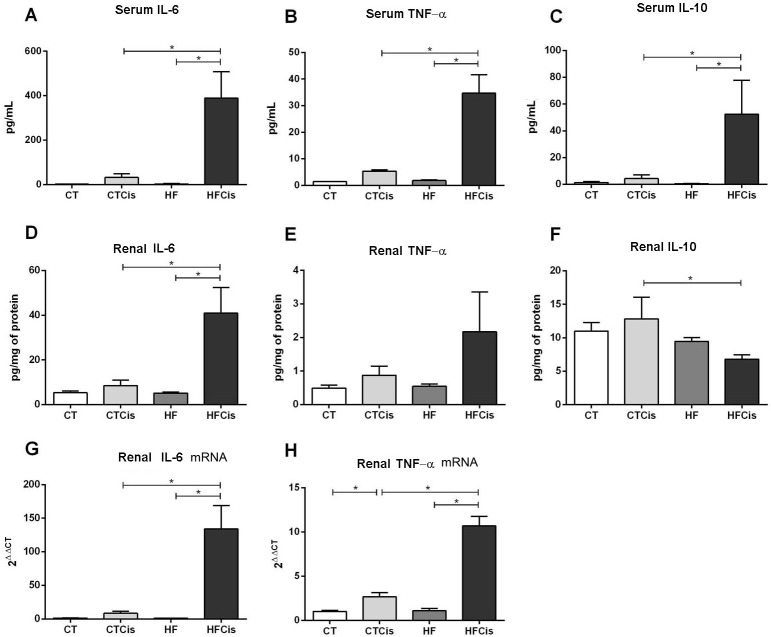
Inflammatory profile. A-C: Serum levels of IL-6, TNF-α and IL-10; D-F: Renal protein levels of IL-6, TNF-α and IL-10. The serum and renal protein levels were determined by using the multiplex immunoassay (ELISA). G-H: Renal gene expression of IL-6 and TNF-α was measured by real-time PCR, and the mRNA levels were normalized to the housekeeping gene β-actin and calculated using the 2^ΔΔCT^ method. The data are expressed as the mean ± SEM, n = 6 per group, *p<0.05.

HF diet animals presented signs of systemic inflammation with increased serum levels of the proinflammatory cytokines IL-6 (65%) and TNF-α (28%) with decreased levels of the anti-inflammatory cytokine IL-10 (60%). Although these changes were not statistically significant, they may have a biological relevance and thus these results were numerically presented in Table 3 and in Fig 7 together with cisplatin treated groups. The inflammatory markers IL-6, TNF-α, and IL-10 in renal tissue were unchanged in the HF animals (Table 3).

**Table 3:** Inflammation markers.

	Parameters	CT	HF
		(n = 6)	(n = 6)
	IL-6 (pg/mL)	1.66±0.40	2.75±1.27
**Serum**	IL-10 (pg/mL)	1.38±0.75	0.56±0.05
	TNF-α (pg/mL)	1.44±0.04	1.84±0.25
	IL-6 (pg/mg protein)	5.30±0.82	5.20±0.36
**Kidney**	IL-10 (pg/mg protein)	11.01±1.28	9.45±0.60
	TNF-α (pg/mg protein)	0.49±0.09	0.55±0.07

Parameters were measured in serum and in renal tissue obtained from CT and HF animals receiving vehicle.

Fig 7 shows the effects of cisplatin on systemic and renal inflammation markers. The effects of cisplatin were discrete on systemic inflammation in CT animals, but a significant rise in the renal content of TNF- α mRNA (Fig 7H). In contrast, there was an overreaction in obese animals to cisplatin with impressive increases in the serum levels of IL-6 (400-fold) and TNF-α (40-fold). Serum IL-10 was also significantly elevated in the HFCis group (Fig 7A–7C). A similar inflammatory profile was observed in the renal tissue of the HFCis group, with higher levels of IL-6 and TNF-α proteins (Fig 7D and 7E) and gene expression (Fig 7G and 7H). In contrast to the systemic levels, the levels of intrarenal IL-10 were reduced in the HFCis group compared with the CTCis group (Fig 7F), contributing to renal inflammation.

## Discussion

It is well established that obesity is considered to be an independent risk factor for kidney disease, even in the absence of diabetes or hypertension [8] and the renal changes observed in the present study suggest that a moderate degree of obesity imposed during childhood may predispose these individuals to precociously develop some degree of renal dysfunction during adulthood. Moreover, the superimposed injury induced by cisplatin caused an overreaction of the obese mice compared with non-obese animals resulting in devastating systemic and renal effects.

To understand the exacerbated effect of cisplatin in obese mice, is important to observe the systemic and renal responses evoked by the obesity induced during the childhood. Animals fed a HF diet within the first week after weaning presented visceral fat accumulation with higher body mass gain compared to animals fed a standard diet. The body mass gain was compatible to a moderate degree of obesity based on the criteria considered for rodents [20–22]. HF animals presented neither diabetes nor hypertension and only a mild elevation in the proinflammatory cytokines IL-6 and TNF-α in serum, that, in spite of non-significant may be indicative of ongoing systemic inflammation process.

In spite of these discrete systemic alterations, obese mice presented many of the renal manifestations related to obesity, including the glomerular hyperfiltration, sodium retention and expansion of Bowman's space. The hyperfiltration was observed in the absence of the typical obesity-related glomerular hypertrophy [23–25]. The reduction in the FENa may have contributed to the increased renal blood flow and the subsequent increase in the glomerular filtration rate. Expansion of Bowman’s space is often observed in the kidneys of obese animals [25–27] and it is probably related to the marked glomerular hyperfiltration [26]. In spite of the absence of histologically evident glomerulopathy, obese mice presented proteinuria, an important sign of glomerular injury. Podocyte dysfunction is related to proteinuria in obese patients [28] and mice [25]. Accordingly, we found a downregulation of nephrin and podocin expressions in obese mice kidneys that certainly contributed to podocyte injury and to proteinuria. Despite the lack of renal inflammation, kidneys from obese mice showed signs of oxidative stress characterized by an upregulation of gp91phox, that in turn, may have to contributed to suppress the nephrin gene [29, 30] in the present model of obesity.

The deleterious renal effects of cisplatin are well known, however, the AKI induced by this chemotherapeutic agent was much more severe in obese animals. This result is relevant considering the strong relationship between cancer and obesity [17, 18] and suggests that obese patients can be more sensible to the nephrotoxic effects of cisplatin. In fact, obese mice presented visible signs of illness such as lethargy and inability to eat compared with non-obese animals. Thus we decided to evaluate and euthanize animals 72 hr after cisplatin, although the peak of cisplatin-induced renal injury in mice and rats occurs 96 hr after cisplatin administration [31]. While the obese mice presented very severe renal inflammation, oxidative stress and AKI, non-obese animals showed lower degree of inflammation and oxidative stress, probable due to the shorter period of evaluation. After 72 hr, the glomerular filtration rate decreased by 31% in the CTCis group and, in spite of non-significant, it resulted in a significant urea accumulation. In contrast, an impressive decline of 92% in the creatinine clearance was observed in the HFCis group, resulting in exaggerated serum creatinine and urea accumulation in obese animals. These results clearly show a higher susceptibility of the obese mice to develop more severe AKI compared to non-obese animals.

Vascular and proximal tubular injury play a major role in cisplatin-induced nephrotoxicity [13]. Indeed, the cisplatin-induced tubular damage leading to apoptosis and acute tubular necrosis was found in CTCis mice, however, both cell death mechanisms were also exacerbated in obese animals, that presented extensive tubular cell death resulting in excessive loss of sodium and potassium in the urine. Despite the increased potassium excretion, obese animals presented sublethal hyperkalemia probably because of glomeruli with minimal filtration capabilities.

Oxidative stress and renal tissue inflammation are associated with cisplatin-induced nephrotoxicity [32, 33]. Although non-obese mice presented discrete alterations in the markers of inflammation and oxidative stress in response to cisplatin, obese animals were much more susceptible. Together with the greater intrarenal inflammation, obese mice presented a severe profile of systemic inflammation characterized by a vigorous elevation in the circulating levels of IL-6 and TNF-α. Interestingly, obese mice showed a rise in the anti-inflammatory IL-10, probably as a response to compensate the exaggerated release if IL-6 and TNF-α, but this hypothesis needs to be investigated. Thus, the presence of obesity caused an exaggerated inflammatory response to cisplatin challenge with a heightened oxidative stress, resulting in major organ damage. The presence of baseline low levels of systemic inflammation in obese animals may contribute to the overreaction to cisplatin, which was probably mediated by a sensitization mechanism. Moreover, the extensive degree of cell death observed in the obese kidneys certainly contributed to the increased renal inflammation developed by obese mice and thus for the rapid deterioration of renal function and more sever AKI after cisplatin administration.

Obese mice presented an increase in visceral fat mass after cisplatin injection, and the expansion of adipose tissue could contribute to systemic inflammation profile. The increase in visceral fat accumulation after cisplatin administration is an intriguing observation, but this phenomenon has also been detected in patients with testicular cancer shortly after administration of cisplatin-based chemotherapy, with an increase in liver triglyceride content mainly observed in obese patients [34]. Currently, there is no explanation for the adiposity expansion after cisplatin treatment, but based on the literature data, we speculate that aldosterone could play a role in this behavior since 1) adipose tissue expresses mineralocorticoid receptors [35, 36]; 2) aldosterone induces adipogenesis [37, 38] and 3) cisplatin is able to increase aldosterone levels [39, 40].

In summary, the results showed that this model of high-fat diet consumption in post-weaned mice induced a moderate degree of obesity that was characterized by low-grade of systemic inflammation with glomerular hyperfiltration. The renal changes found in the young obese mice suggest that the development of mild obesity during childhood may predispose these individuals to precociously developing some degree of renal dysfunction in adulthood. Obese mice presented a greater susceptibility to the toxic effects of cisplatin and developed more severe AKI than the non-obese mice, which was mediated by an exaggerated systemic and renal inflammatory response. In conclusion, obesity predisposed young adult mice to develop much more severe acute kidney injury induced by cisplatin.
